# A new approach to three-dimensional microstructure reconstruction of a polycrystalline solar cell using high-efficiency Cu(In,Ga)Se_2_

**DOI:** 10.1038/s41598-024-52436-2

**Published:** 2024-01-23

**Authors:** Chang-Yun Song, Matthias Maiberg, Heiko Kempa, Wolfram Witte, Dimitrios Hariskos, Daniel Abou-Ras, Birgit Moeller, Roland Scheer, Ali Gholinia

**Affiliations:** 1 Institute of Physics, Martin Luther University Halle-Wittenberg, Von-Danckelmann-Platz 3, 06120 Halle (Saale), Germany; 2 Zentrum für Sonnenenergie- und Wasserstoff-Forschung Baden-Württemberg (ZSW), Meitnerstr. 1, 70563 Stuttgart, Germany; 3 Helmholtz-Zentrum Berlin für Materialien und Energie GmbH, Hahn-Meitner-Platz 1, 14109 Berlin, Germany; 4 Institute of Computer Science, Martin Luther University Halle-Wittenberg, Von-Seckendorff-Platz 1, 06120 Halle (Saale), Germany; 5 Department of Materials, The University of Manchester, Manchester, M13 9PL UK

**Keywords:** Characterization and analytical techniques, Photovoltaics, Engineering, Materials science

## Abstract

A new method for efficiently converting electron backscatter diffraction data obtained using serial sectioning by focused ion beam of a polycrystalline thin film into a computational, three-dimensional (3D) structure is presented. The reported data processing method results in a more accurate representation of the grain surfaces, reduced computer memory usage, and improved processing speed compared to traditional voxel methods. The grain structure of a polycrystalline absorption layer from a high-efficiency Cu(In,Ga)Se_2_ solar cell (19.5%) is reconstructed in 3D and the grain size and surface distribution is investigated. The grain size distribution is found to be best fitted by a log-normal distribution. We further find that the grain size is determined by the [Ga]/([Ga] + [In]) ratio in vertical direction, which was measured by glow discharge optical emission spectroscopy. Finally, the 3D model derived from the structural information is applied in optoelectronic simulations, revealing insights into the effects of grain boundary recombination on the open-circuit voltage of the solar cell. An accurate 3D structure like the one obtained with our method is a prerequisite for a detailed understanding of mechanical properties and for advanced optical and electronic simulations of polycrystalline thin films.

## Introduction

For understanding the properties and growth mechanisms of polycrystalline thin films, an analysis of the microstructure is essential. Quantities such as grain size, size distribution, grain boundaries, and grain orientations are critical parameters to characterize thin films and are closely related to electromagnetic properties^1–5^. However, most studies have relied on traditional 2D methods such as scanning electron microscopy (SEM)^6^, X-ray diffraction (XRD), transmission electron microscopy (TEM)^7^ and electron backscatter diffraction (EBSD)^4^. These methods provide only sectional information. In order to obtain comprehensive information on the volume and surface of grain it is essential to use three-dimensional (3D) analysis techniques. Therefore, SEM techniques^8^ including EBSD^2,9^ have been applied for the analysis of 3D structures in addition to optical microscopes^7^. The serial sectioning with SEM is typically obtained through techniques such as mechanical milling^10^, broad ion beam milling (BIB)^11^, focused ion beam milling (FIB)^9,12^ plasma-FIB milling^13^{FormattingCitation}{FormattinCitation}, and femtosecond laser ablation^14^, all of which are destructive techniques.

X-ray methods such as 3D X-ray diffraction (3DXRD)^15^ and diffraction computed tomography (DCT)^16^ are non-destructive techniques and allow for the measurement of 3D structures. However, the resolution of 3D X-ray CT methods are in microns and much poorer compared to electron microscope serial sectioning 3D EBSD that are in tens of nanometers^15–19^. Despite the development of various imaging methods, 3D structure analysis is not widely used because of the complexity of 3D data acquisition and processing^9^. A common processing method of representing 3D structure data is the voxel method, which involves representing all data points in a single cube^14,20^. However, this approach can result in an uneven surface structure and is only suitable for cases where the grain size is significantly larger than the resolution^11,21^.

Due to the lack of accurate 3D models research on high-efficiency solar cell films, where the size of the crystal grain is a limiting factor, so far has mainly relied on two-dimensional methods^4^. Only a very small number of 3D solar cell structures have been reported^22^. Moreover, no 3D simulations of optoelectronic device properties based on experimental structure models are available to date^4^. This is particularly true for an accurate 3D structure of a Cu(In,Ga)Se_2_ (CIGSe) thin film in a working solar cell.

In this study, we present an efficient method for reconstructing a polycrystalline CIGSe absorber thin film of a high-efficiency solar cell. We obtained serial sections of the CIGSe absorber layer through FIB milling and performed EBSD measurements on each section to acquire 2D grain orientation maps. The 3D microstructure was then reconstructed through data processing using a self-written computer program. To this end, an alternative method to the voxel method was developed to achieve more accurate grain surfaces, i.e., more accurate grain boundaries. Furthermore, we investigate the accuracy and efficiency of the proposed 3D reconstruction method and analyze the reconstructed 3D data in conjunction with glow discharge optical emission spectroscopy results in order to study the grain structure and growth mechanism of CIGSe^23,24^. Using the analyzed information, we applied the constructed CIGSe model to 3D solar cell simulations.

## Experimental and process flow

### Sample preparation

In this work, a CIGSe solar cell with a high power conversion efficiency of 19.5% was used. The sample was fabricated at ZSW by co-evaporation of CIGSe on molybdenum-covered glass substrates with an average [Ga]/([Ga] + [In]) ratio (GGI) of 0.35. The polycrystalline CIGSe absorber with tetragonal structure and a thickness of 2.8 µm was deposited with a standard three-stage process in a static deposition chamber. During the CIGSe growth process at elevated temperatures, Na and partially K were provided from the glass substrate to the absorber. At the end, the absorber underwent an in-situ CsF post-deposition treatment under Se atmosphere without breaking the vacuum. A solution-grown CdS buffer layer was deposited on top, followed by rf-sputtered Zn_0.85_Mg_0.15_O and rf-sputtered ZnO:Al as the transparent front contact. The cell, with a total area of 0.5 cm^2^, has Ni/Al/Ni grid fingers on top and was fabricated without an anti-reflective coating (ARC), thereby lacking an absolute current gain of 1–1.5%. With an ARC, a cell efficiency of above 20% would be expected.

### Thin-film characterization as input for 3D model

**Figure 1:**
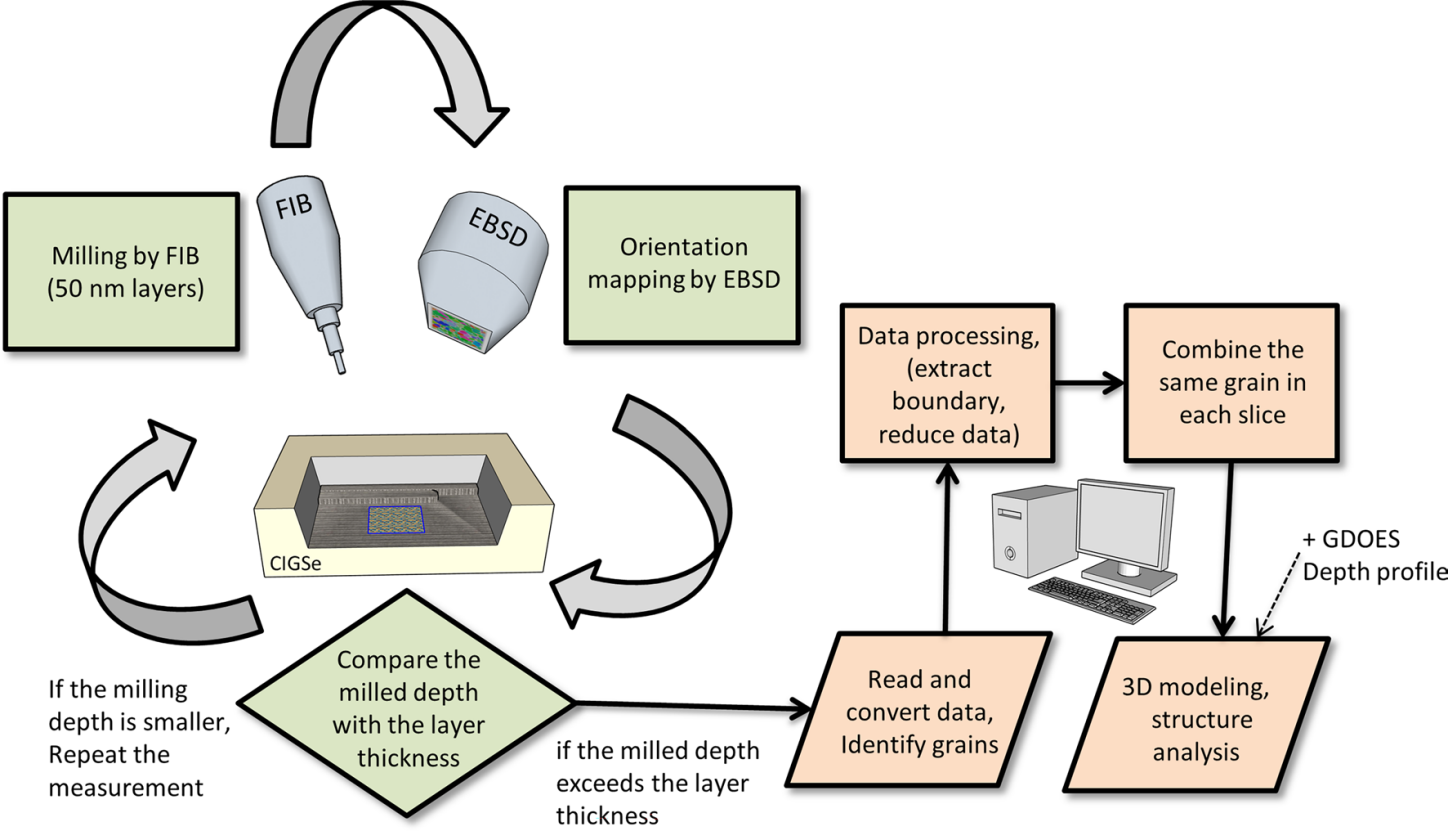
A schematic flowchart illustrating the process of generating 3D structures using EBSD data. Cross-sectional slices are created using FIB and repeatedly measured. The resulting diffraction pattern is converted into orientation and grain ID data for each coordinate. The data is then efficiently processed to generate the 3D structure and conduct structural analysis.

The 3D EBSD measurement was performed using the latest Helios™ 5 Laser PFIB from Thermo Scientific. The slice and view software was used to automatically gather the data by a serial sectioning method with a top down geometry to minimize charging from the glass substrate during milling and EBSD. A fiducial mark was milled to the edge of the sample to help the alignment of the region of interest during the automatic run. The Xe plasma focused ion beam (PFIB) was set to 30 kV accelerating voltage and 15 nA beam current and used to mill 80 slices with 50 nm slice thickness. The electron beam for the EBSD analysis was set to 20 kV accelerating voltage and 1.6 nA probe current. The EBSD analysis was done using the Symmetry EBSD camera, AZtec and AZtecCrystal software from Oxford Instruments plc. The EBSD mapping area was 200 × 200 pixels at 50 nm step size and the Kikuchi pattern resolution was set at speed 2 which has a pixel resolution of 156 × 128. The EBSD mapping was done at more than 600 Hz to ensure good indexing rate above 70%, which is as expected for such a microstructure with a fine grain size of approximately 1 μm equal circle diameter. The overall procedure of the experiment is depicted in Fig. 1.

The thickness and the chemical composition of the CIGSe absorber layer, including the GGI was determined by GDOES measurements using a GDA 750 HR instrument by Spectruma Analytik. The analysis was conducted by measuring the depth profiles of the chemical composition of the sample, and the WinGDOES software was used to convert the measured emission line intensity data into components and their concentrations.

## Results and discussion

### Modeling process

#### Alignment

**Figure 2:**
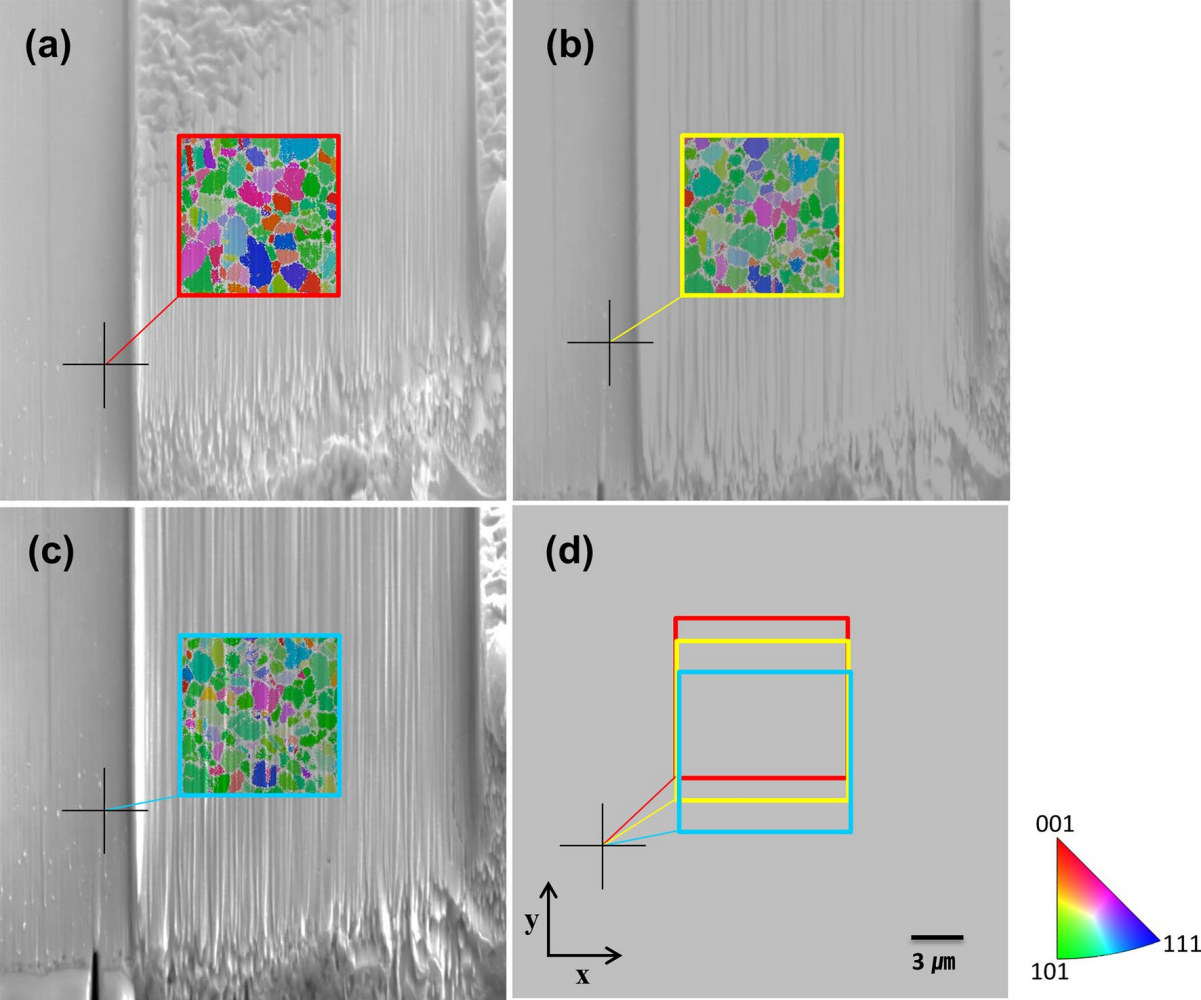
EBSD inverse pole figure map (colored, in the center) and corresponding SEM images (gray, in the background) illustrate the microstructural analysis of the CIGSe absorber layer at varying depths. The series of images captures the evolution in grain orientation and size with increased depth. At 0.55 μm depth, (**a**) presents the combined EBSD and SEM data, similarly (**b**) at 1.65 μm, and (**c**) at 2.00 μm. In (**d**), the EBSD measurement locations, identified by reference points (black crosses) from the SEM images, display slight xy plane wobbles and y-axis shifts with depth, underscoring the importance of alignment.

**Figure 3:**
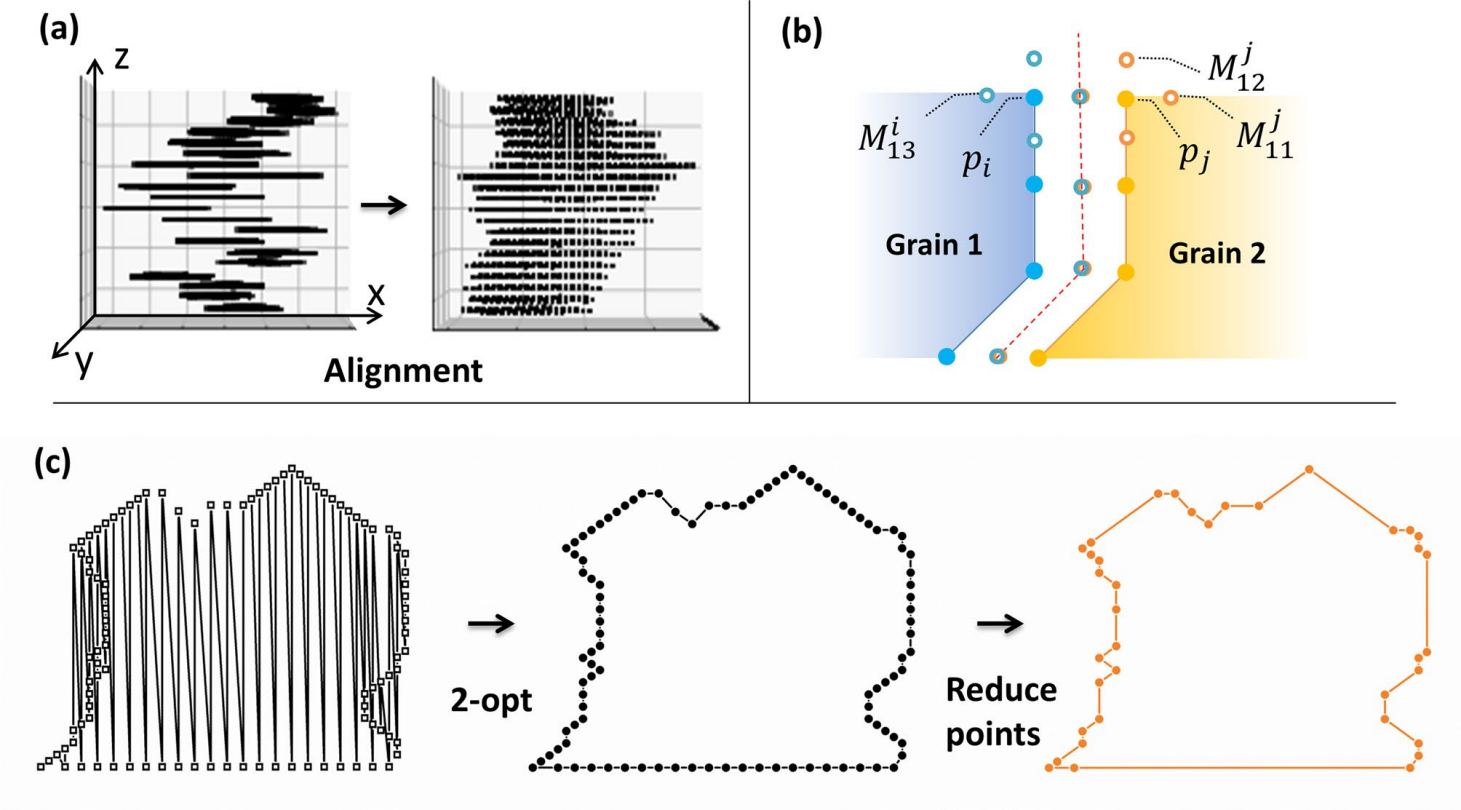
Examples and results of each data processing method. (**a**) Shows the original data (left) and the grains after correcting X and Y axis translation errors through alignment (right), with the data appearing closer to actual grain shape. (**b**) Example of the ‘out-line’ method, original data (solid) and expanded data (open) and the newly formed grain boundary (dotted line). (**c**) Data aligned into grain shapes by the ‘2-opt’ process and data with significantly reduced points while maintaining the same shape through the 'reduce-point' process (solid orange).

During 3D data acquisition, translational errors can occur due to sample movement or charging, Fig. 2a shows the measurement results at a depth corresponding to 0.55 µm in the 11th slice, while Fig. 2b,c show the results at depths 1.65 and 2.0 µm in the 33rd and 40th slices, respectively. Reference points (black crosses) were set using specific particles observed in the surrounding area, outside the region to be cut. As shown in Fig. 2d, the field of the EBSD measurement area varies with the milling depth after matching the reference point of each EBSD measurement slice. The translational drift, which can be observed in Fig. 3a left, can be caused by sample charging or movement during data acquisition. It is clear that proper alignment is crucial for obtaining accurate 3D data^9,10^. Thus, an initial preprocessing step based on the SEM images was used for alignment. The whole set of SEM images consisted of 79 images with a resolution of 630 × 474 pixels which refers to a physical resolution of approximately 52.939 × 39.830 µm, therefore 84 nm per pixel in x and y direction.

Pixel shift calibration was performed by using each pair of consecutive images and the parameters of a translational alignment were estimated. To this end, we implemented a registration operator in MiToBo^25^ based on the MPI-CBG stitching library developed by Stephan Preibisch and others^26^. Thus, pairwise image translations are estimated based on phase correlation in Fourier space of two given images. The resulting pixel shifts in x and y direction are given with subpixel accuracy. Once the translational shift between two images is known the translation parameters can be concatenated to register each image with regard to the first image of the sequence. As a final step, the pixel shifts from the calibration of the SEM images were converted to micrometers so that the estimated model parameters could be applied to other measurement data to compensate for the translational drifts. However, the FIB milling process also introduces a height discrepancy between the reference point used for alignment and the location at which EBSD measurements are taken. This results in linear tilt errors, which were corrected by additional alignment using the center point of an arbitrary large grain^21^. The process of the complete alignment can be seen in Fig. 3a.

#### Two-dimensional data process

Consider an EBSD image which gives orientation information for each pixel in a grain. In order to facilitate efficient computer memory use and data processing, it is sufficient to represent each grain by its contour pixels and discard the interior pixels. Therefore, the pixel data of the grains’ interiors were removed from each 2D cross-section, and the grains were constructed using only their surface pixels.

To generate the outline of a grain (‘out-line’ algorithm), we used expanded data that include coordinates corresponding to double the measurement resolution instead of using the raw measurement positions. The method is depicted in Fig. 3b where the open points depict the expanded data introduced to represent the grain boundary, which simultaneously belongs to the contours of both grains. This allowed us to create shared boundaries of neighboring grains and prevent them from being separated due to an EBSD measurement gap. The detailed algorithm for generating the grain outline is described below.

1 $$\begin{aligned} & P_{i} = \left( {x_{i} ,y_{i} } \right) \to M^{i} \\ & M^{i} = \left[ {\left( {x_{i} + \frac{{g_{x} }}{2},y_{i} } \right), \left( {x_{i} ,y_{i} + \frac{{g_{y} }}{2}} \right), \left( {x_{i} - \frac{{g_{x} }}{2},y_{i} } \right), \left( {x_{i} ,y_{i} - \frac{{g_{y} }}{2}} \right)} \right] \\ & {\text{M}}^{i} \cap {\text{M}}^{j} \to {\text{new }}\,{\text{point }}\left( {{\text{P}}^{\prime } } \right) \\ & {\text{Delete}}:({\text{M}}^{i} \cap {\text{M}}^{j} )^{C} \\ \end{aligned}$$

here the original data point P_i_ expands into the set of coordinates M^i^ by doubling the resolution of the x-axis (g_x_) and y-axis (g_y_) respectively. M^j^ represents the set of coordinates generated from the point data P_j_ of the neighboring grains. If M^i^ and M^j^ are compared and found to be in contact, meaning they share the same coordinates, a new point (P′) indicating the grain boundary is generated. This procedure is necessary to avoid empty space between grains. After replacing P_i_ as described above, we obtain a new set of position data now referred to as P'.

Since the ‘out-line’ algorithm produces grain boundaries as x, y coordinates based on the electron beam scanning (see Fig. 3c left), the data must be sorted for the program to recognize the grain's contour. To this end, we employed the 2-opt algorithm^27^ in order to rearrange the point data (see Fig. 3c middle). The 2-opt algorithm is a mathematical optimization technique developed to address the traveling salesman problem. It creates a new path by exchanging two points, ensuring that the path has no crossover and has a shorter distance than the existing path. As shown in Fig. 3c—the ‘2-opt’ grain is properly rearranged through the process. This process continues until the sum of all paths reaches a minimum according to Eq. (2).

2 $$Paths = min\left[ {\sum\nolimits_{(i = 0)}^{N} {d\left( {P_{i}^{\prime } ,P_{(i + 1)}^{\prime } } \right)} + d\left( {P_{N}^{\prime } ,P_{0}^{\prime } } \right)} \right]$$

here P′_i_ is the i-th point, d(P′_i_, P′_j_) is the path length between P′_i_ and P′_j_ and N is the total number of data^27^.

The data were further condensed through the ‘Reduce-points’ process as shown in Fig. 3c right. Since the EBSD data are given on a quadratic pattern, the angle *θ* between two vectors formed by three consecutive points can be 0, 45, or 90 degrees. It can be calculated from

3 $$\begin{aligned} & \theta = \cos^{ - 1} \left( { \frac{{\left( {P^{\prime}_{i - 1} - P^{\prime}_{i} } \right) \cdot \left( {P^{\prime}_{i} - P^{\prime}_{i + 1} } \right)}}{{\left\| {P^{\prime}_{i - 1} - P^{\prime}_{i} } \right\| \cdot \left\| {P^{\prime}_{i} - P^{\prime}_{i + 1} } \right\|}}} \right) \\ & if \; \theta = 0 \to Delete \,point \\ \end{aligned}$$

when $\theta$= 0 within a certain tolerance, the points are connected by a straight line and P′_i_ can be removed in order to reduce the size of the data while maintaining the shape of the grain.

#### Grain identification

The processed 2D grain data is grouped and identified as a single grain. Center position, grain size, Euler angle, and Euler spread are used to identify the same grain in subsequent EBSD slices. More weight is given to Euler angle and spread, which represent the orientation information, rather than size or position information. Small grains with a thickness of less than 100 nm that cannot be confirmed to be connected across two or more slices are excluded from the model. After identification, each grain is assigned a unique ID, and data of the same grain from different slices are grouped together by means of position, Euler angles, and spread.

#### 3D reconstruction

**Figure 4:**
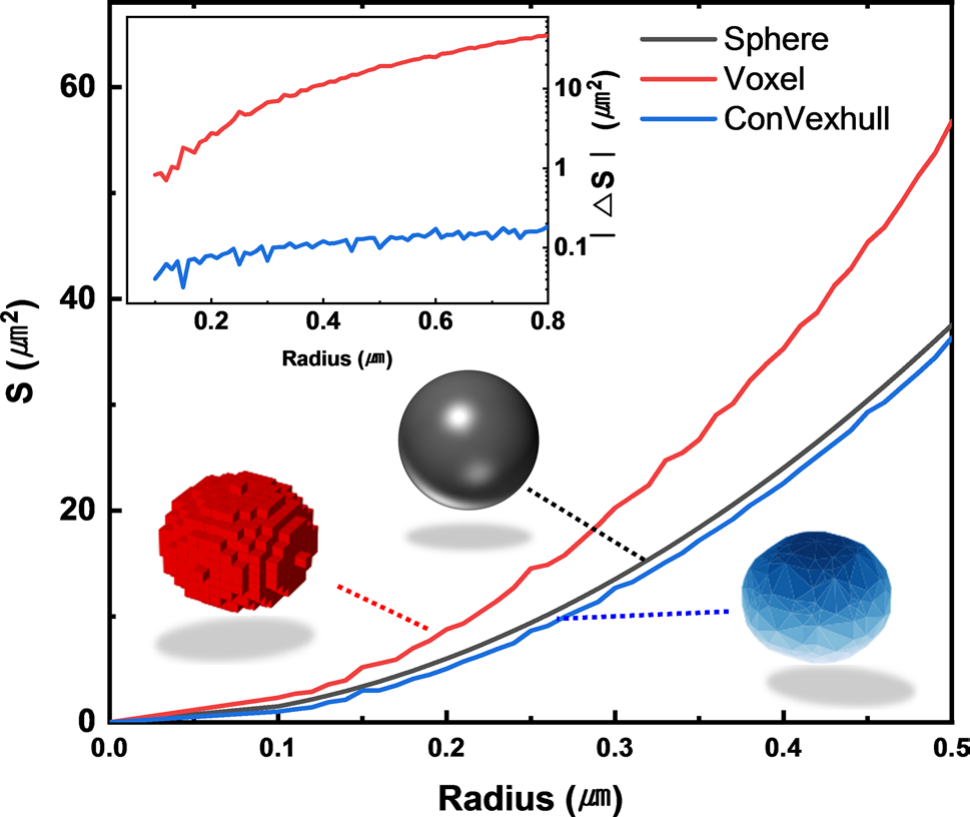
Comparison of the sphere's surface area of a sphere S as calculated by the voxel (red line) and convex-hull (blue line) methods against the actual surface area (black line) as a function of radius. Calculations were performed at a 50 nm resolution, akin to the EBSD measurement resolution. The inset quantifies the absolute difference in surface area between the actual sphere and the modeled spheres, indicating a closer match with the convex-hull method.

The 2D slices of each grain were then extended to 3D using the convex-hull algorithm^28^. There are several reasons for using the convex-hull method, which is considered more complex than the voxel method commonly used when forming a 3D structure^29,30^. Firstly, it provides a more realistic surface area value. This is particularly evident when grains are small in volume compared to the resolution. Figure 4 illustrates the surface area of a real sphere according to the radius R, and the surface area S of the model created by the two methods, voxel and convex-hull. The convex hull method consistently yields values similar to the actual surface area, while the voxel method increasingly deviates with increasing sphere radius. Having an accurate surface area value is important when studying properties like compositional inhomogeneity or recombination mechanisms, which are affected by grain boundaries. Secondly, unlike the voxel method, which expresses all measured points in a cube form, the convex-hull method reduces the number of data by not using internal information, resulting in a reduction in the running time for modeling or simulation.

**Figure 5:**
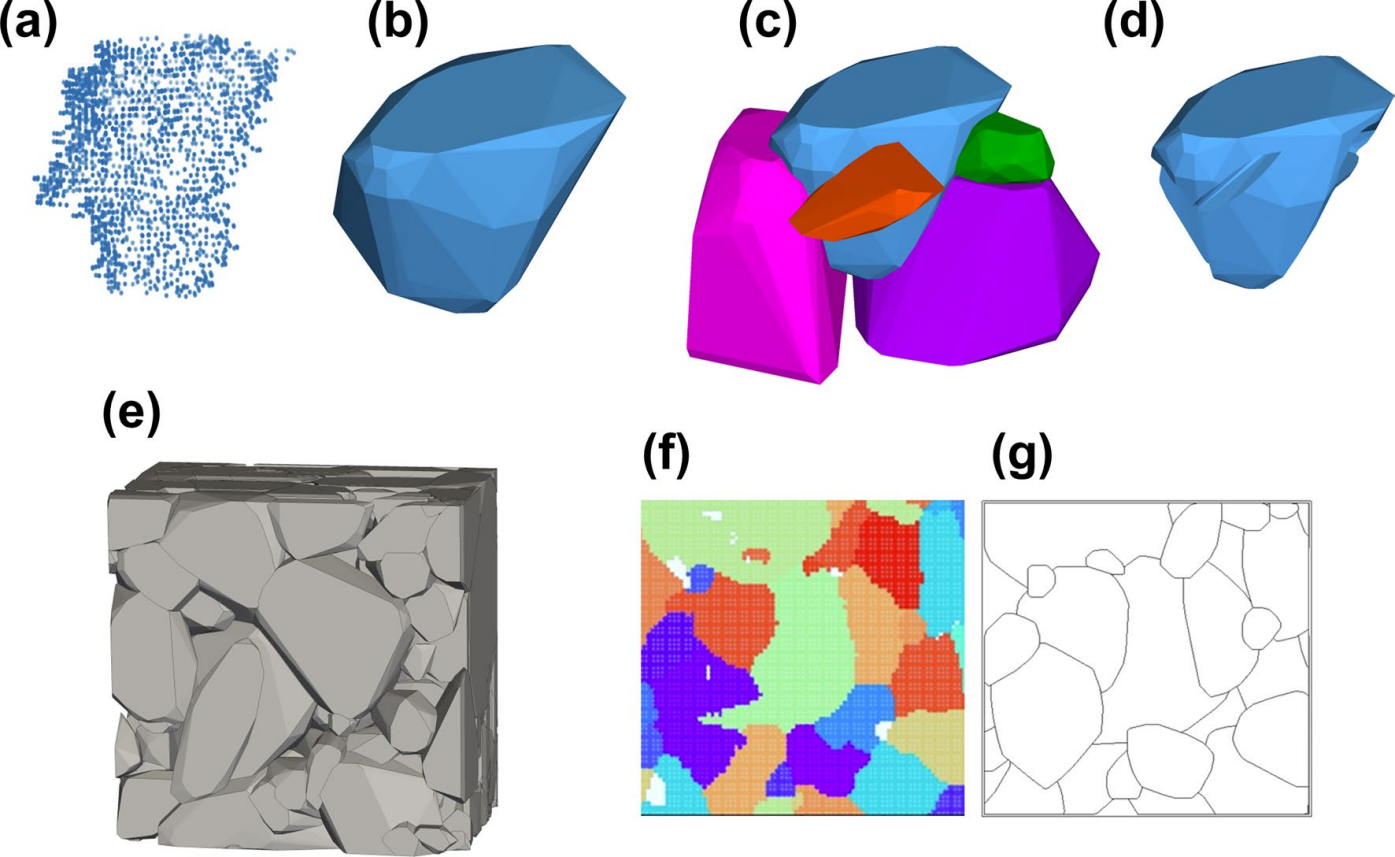
(**a**–**d**) depict the steps to transform 3D data points of a single grain into a 3D model using the convex-hull method (see text for details): (**a**) displays the raw data points, (**b**) the convex-hull generated shape, (**c**) the placement of adjacent grains for context, and (**d**) the refined shape after eliminating overlaps. (**e**) shows the reconstructed 3D volume of a 4 × 4 × 3 µm^3^ CIGSe sub-sample. A comparison between (**f**) the EBSD raw data from a random slice and (**g**) a corresponding cross-section of the reconstructed volume reveals a high similarity, with only minor details differing.

It is worth noting that the convex-hull algorithm simplifies the data by covering all points (Fig. 5a) within the interior of the grain, leaving only the outermost shell. In this process, detailed information of each grain is primarily lost, as shown in Fig. 5b. However, if the overlapping part is removed, considering the neighboring grains (Fig. 5c), the details of each grain that were lost through the convex-hull algorithm reappear, as shown in Fig. 5d. The structure becomes more accurate the greater the number of neighboring grains considered. Although minor structural information may be lost, the structure is reconstructed enough to be considered as the ‘actual grain structure’. Figure 5e shows an image of the finished 3D grain structure of the CIGSe film. Figure 5f,g provide a comparison of the EBSD raw data of a randomly chosen slice and the cross-section of the reconstructed 3D model at the same position. It can be seen that, despite minor differences, the overall grain structure is well represented by the 3D reconstruction.

The number of data points reduced through each process step is summarized in Table 1. In 2D data processing (‘Out-line’ and ‘Reduce-point’), the total amount of data was reduced to 23.5%. The final data that has been completed up to the 3D ‘convex-hull’ process is only 3.3% compared to the raw data, which is an excellent performance considering that more data is generally required when 2D data is expanded to 3D.

**Table 1:** Number of data points, reduction rate and percentage of rate with raw data amount after each process step.

Method	Number of points	Reduction [%]	Total rate [%]
Raw data	352,000	–	100
Out-line	160,465	54.4	45.6
Reduce-point	82,771	48.4	23.5
Convex-Hull	11,634	85.9	3.3

‘Raw data’ represents the data before processing, ‘Out-line’ extracts only the boundary information, ‘Reduce-point’ removes unnecessary data on a straight line, and ‘Convex-hull’ simultaneously connects and simplifies all data in a three-dimensional space.

### Geometric analysis

#### Surface area distribution analysis

**Figure 6:**
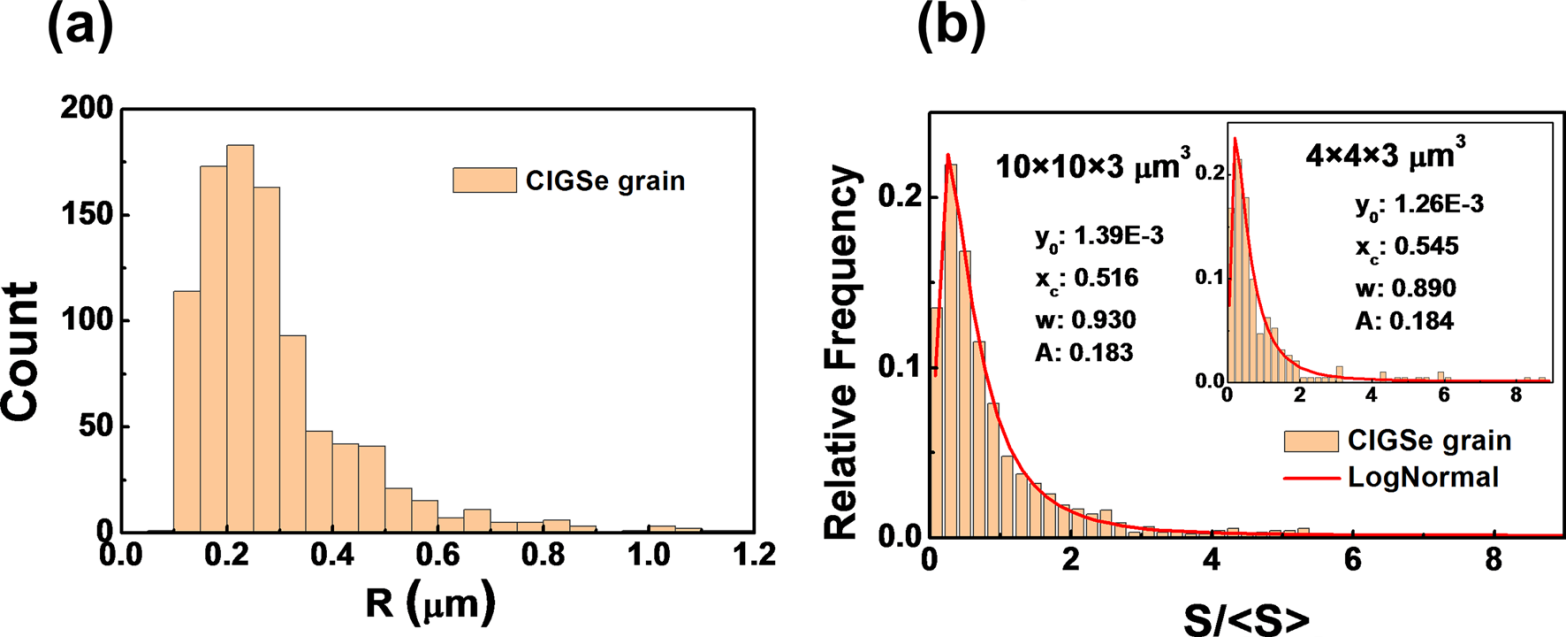
Analysis of grain structure in the CIGSe layer based on 3D reconstruction. (**a**) Histogram of the equivalent sphere radii R for grains, indicating that a significant number of grains have a size of approximately 0.3 μm. (**b**) Normalized surface area distribution S/<S> of grains for the 10 × 10 × 3 μm^3^ sample and the 4 × 4 × 3 μm^3^ sub-sample (inset), with both distributions closely fitting a LogNormal distribution (red line). The similar fitting parameters for the 10 × 10 × 3 μm^3^ and 4 × 4 × 3 μm^3^ indicate that the 4 × 4 × 3 μm^3^ sub-sample is suitably representative of the grain structure.

We will now use the digital representation of the CIGSe grain structure to perform an analysis of the geometric properties of all grains in the analyzed 10 × 10 × 3 µm^3^ volume and their distributions. The distribution of the equivalent sphere radius R, that is, the radius of a sphere with identical volume is shown in Fig. 6a. It can be seen that most of the grains are about 0.3 μm in size, while some grains reach more than 1 μm. However, the proportion of the volume taken up by large grains is larger than that of small grains. In Fig. 6b, the frequency distribution of the surface area S of the grains normalized by the average surface area <S> is displayed. It can be well fitted with a LogNormal distribution

4 $$y = y_{0} + \frac{A}{\sqrt 2 \pi \omega x} exp\left( {\frac{{ - \left( {\ln \frac{x}{{x_{c} }} } \right)^{2} }}{{2\omega^{2} }}} \right)$$

where y_0_ is the offset, A is the area, $\omega$ is the log standard deviation and x_c_ is the center. The fit parameters y_0_, A, ω, and x_0_ can be used to determine the structural similarity of sub-samples of the analyzed volume^31,32^. It can be seen that the fit parameters, hence the grain surface distribution, are very similar for the 10 × 10 × 3 µm^3^ sample and a 4 × 4 × 3 µm^3^ sub-sample. The same analysis has been performed for 8 × 8 × 3 µm^3^ and 2 × 2 × 3 µm^3^ sub-samples (see supplementary Fig. S1). The fitting parameters for the 8 × 8 × 3 µm^3^ sub-sample are similar to the 10 × 10 × 3 µm^3^ sample and a 4 × 4 × 3 µm^3^ sub-sample. Thus, a 4 × 4 × 3 µm^3^, sample volume is sufficient for an appropriate representation of the grain structure in our case. In case of the 2 × 2 × 3 µm^3^ sub-sample, the grain surface distribution significantly deviates. Hence for too small a sample volume, the number of incompletely contained grains cannot be neglected. We therefore conclude, that 4 × 4 × 3 μm^3^ is the minimum size for an appropriate representation of the grain structure in our case. The lateral sample dimensions are more than 10 times the size of the majority of grains and about 4 times the size of the largest grains. We note that choosing a small sample size would reduce computational times for simulation tasks.

#### Relationship between surface area and grain size

**Figure 7:**
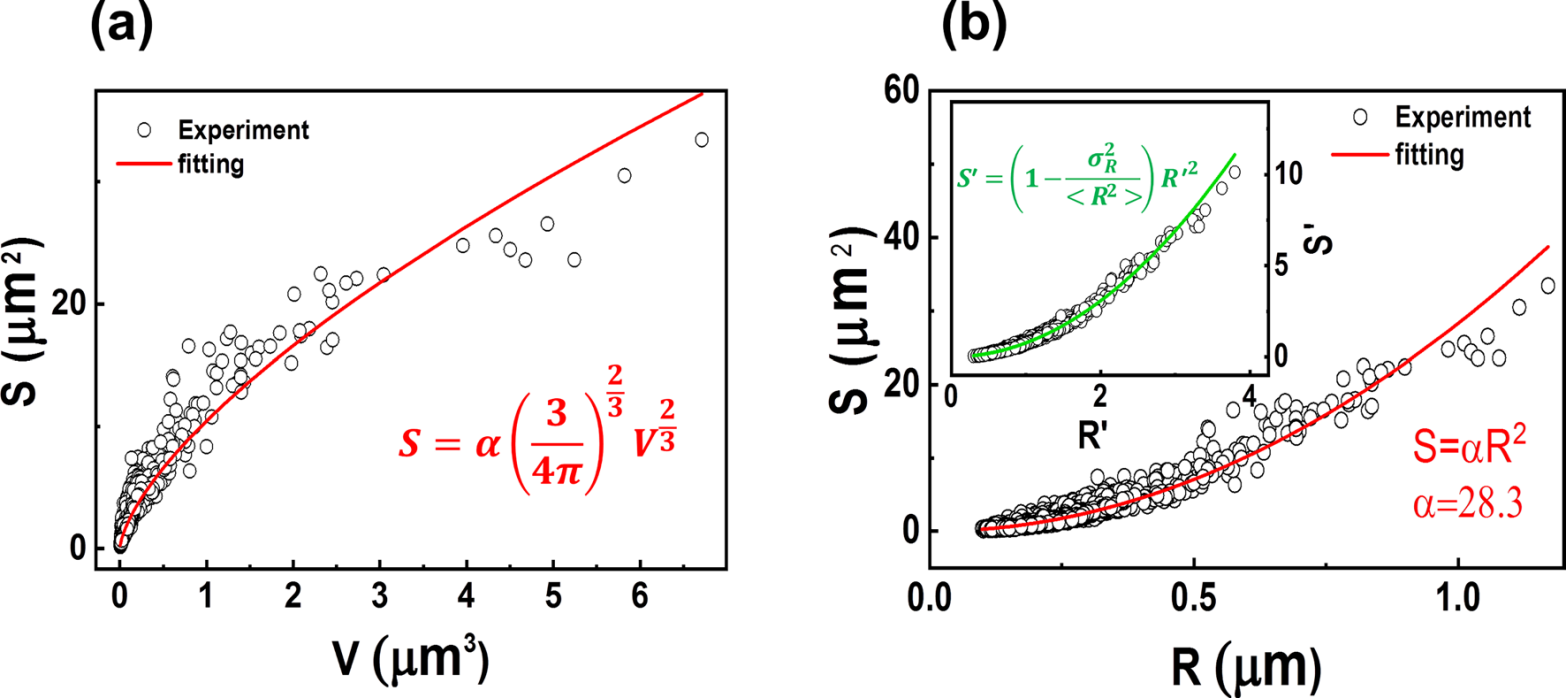
(**a**) The relationship between surface area S and grain volume V, and (**b**) surface area versus grain radius R for CIGSe grains. Open circles indicate experimental data, which align closely with the theoretical relationship (red line). The inset in (**b**) plots the normalized surface area (S′) against the normalized radius (R′), following Eq. (6) (green curve), demonstrating that the relationship between S′ and R′ is governed by the variance in R.

Next, one can ask if there is a relation between the surface area of the grains and their respective grain volume. As shown in Fig. 7a, the surface area increases as the volume (V) of the grains increases, but the rate of this increase gradually diminishes with larger volume. The red line indicates that there might be a relation of the form S ∝ V^2/3^. By utilizing the relationship V ∝ R^3^, where R is again the equivalent sphere radius, we can reformulate this as S ∝ R^2^. At this point, we introduce the shape coefficient α as introduced in references^33,34^. That means, the surface area is expressed as S = αR^2^. The minimum value of α is 4π ≈ 12.6, which corresponds to a sphere with the least surface area of all possible geometrical shapes. As shown in Fig. 7b, the value for our sample is approximately constant at 28.3, except for a few larger grains. This suggests that the CIGSe grains possess a higher surface area to volume ratio compared to spheres. It further suggests that the majority of grains exhibit similar shapes because they have an identical α. This constant value of α may be attributed to the CIGSe growth process. It is known that the films undergoes a recrystallization in the Cu-rich phase of the CIGSe growth process (here the second stage of the 3-stage process)^35^. The constant α value for each grain suggests that this is a homogeneous recrystallization process of the grains in 3 dimensions. Or, in other words, during the recrystallization process each grain grows in volume while keeping its general shape up to a certain limitation imposed by neighboring grains. This shape appears to be far from sphere type. In order to further elucidate the growth phenomena in CIGSe films our newly developed evaluation tool may be future interesting.

To continue, we assume the validity of <S>  = α <R^2^> for the average values of S and R^2^. Then the following expression for the normalized values R′ = R/ <R> and S′ = S/ <S> can be derived.

5 $$S^{\prime} \cdot \left\langle S \right\rangle = \alpha R^{\prime 2} \cdot \left\langle R \right\rangle^{2}$$

6 $$S^{\prime} = \left( {1 - \frac{{\sigma_{R}^{2} }}{{\left\langle {R^{2} } \right\rangle }}} \right) \cdot R^{\prime 2}$$

where ${\sigma }_{R}^{2}$ =  <R^2^>  −  <R> ^2^ is the variance of R. According to Eq. (6), the normalized surface area and the normalized grain size have a directly proportional relation, which does not depend on α. As shown in Fig. 7b inset, this is in good agreement with the experimental data.

#### Grain orientation spread

**Figure 8:**
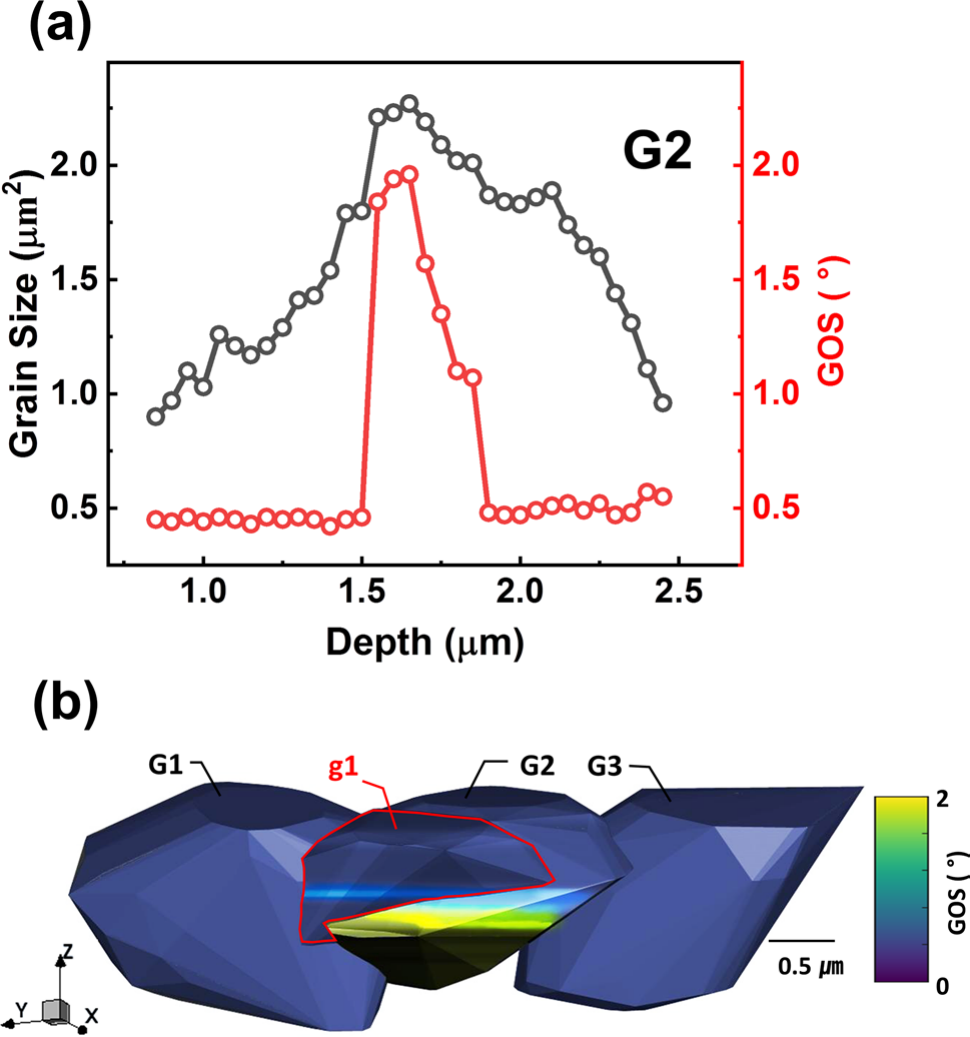
(**a**) Depicts the variation in grain size (black line) and Grain Orientation Spread (GOS, red line) of grain G2 with depth. (**b**) Provides a 3D GOS color map showing the GOS for grain G2 and its neighboring grains. Elevated GOS values at the interface of grain G2 and grain g1 suggest areas potentially subjected to compressive stress; the color bar on the right indicates the GOS values.

In order to study the strain within grains, the grain orientation spread (GOS) was analyzed^36,37^. In each slice, which was characterized by EBSD, the angular difference of the orientation ϑ in each measurement point with the mean orientation <ϑ> was averaged, yielding the GOS value <|ϑ −  <ϑ>|> . It was found that the majority of grains have a low GOS value, with an average value of around 1 degree or smaller, indicating that they have a stable and recrystallized structure. However, some grains g1 and G2, as seen in Fig. 8, have a higher GOS value at specific locations. Although the number of such grains is not large, around 1% of the total, the effect is significant because it occurs mainly in large grains. The GOS increase in G2 reaches up to 2 degrees, which is observed at the intersection points of g1. Interestingly, the GOS of the G2 grain can be seen in Fig. 8a,b, and has a peak value not at the overlap starting point with the g1 grain, but at the position with the largest diameter. While examining Fig. 8a, the stress may appear to be purely tensile, but a more accurate understanding can be obtained from the 3D representation depicted in Fig. 8b. It reveals that the increase in GOS in the middle of the grains is caused by compressive stress generated by a grain overlap that occurred during the growth process (refer to supplementary Fig. S2 for an example involving another grain).

#### Relation between GGI and grain size

**Figure 9:**
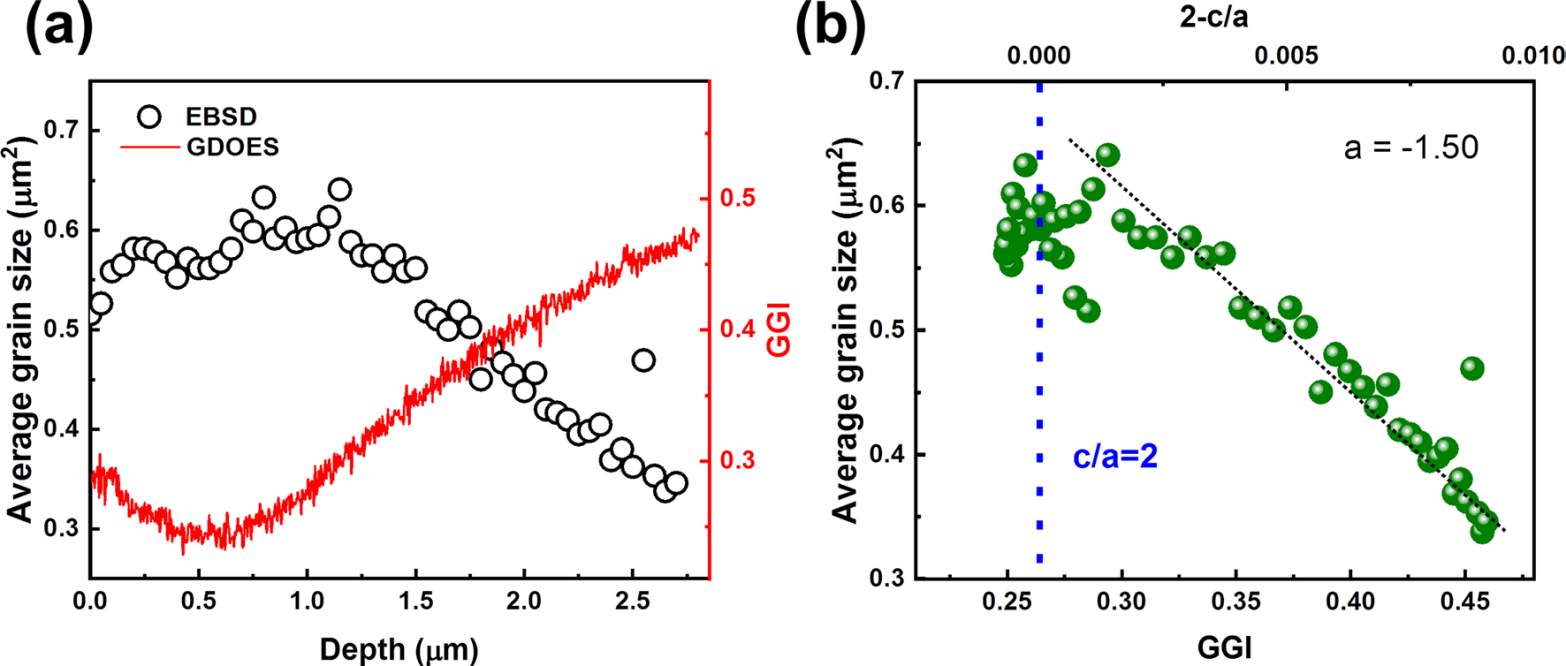
(**a**) Shows the relationship between the average grain size (black open circles) and depth, with the GGI (red line) correspondingly decreasing. As the depth increases, the GGI decreases, and the grain size increases. The average GGI amounts to 0.35. (**b**) Presents the average grain size as a function of both the lattice constant ratio (c/a) and GGI. The grain size reaches a peak at a c/a ratio of 2, as indicated by the vertical blue dashed line, beyond which there is an inverse correlation with increasing GGI, demonstrated by the linear trend line (slope = − 1.5).

GDOES was utilized for compositional analysis along the film depth. This allows to relate the grain structure obtained from EBSD measurements and the chemical composition. Figure 9a shows the GGI as a function of depth as measured from the film surface. The GGI has a minimum at about 0.5 µm and increases towards the front surface and the back contact. Such GGI grading is a common result of the three-stage process of CIGSe deposition^38,39^. Additionally, Fig. 9a gives the average grain size as a function of depth. The depth profile of average grain size (grain-cross section area) was calculated from the total EBSD measurement area in each slice divided by the number of grains in this slice. Comparing grain size and GGI, it is found that the grain size is large at positions of small GGI. A plot of GGI versus grain size (Fig. 9b) gives an approximate linear behavior (correlation coefficient − 0.92) with a slope of − 1.50 µm^2^.Thus, GGI and grain size exhibit an inverse correlation.

Previous studies have investigated various CIGSe films and have also found an inverse relationship between the average grain size and GGI^40,41^. Notably, Ref.^40^ indicates that there exists a direct proportionality between the GGI and the lattice parameter ratio, (c/a). By leveraging this established proportionality, a relationship of (c/a = 2.0125–0.047 × GGI) has been inferred to convert the measured GGI values into (c/a) ratios. These converted ratios are plotted on the upper x-axis of Fig. 9b. It can be observed that as GGI decreases, the grain size increases linearly until it approaches a value close to 2, where it reaches a peak.

The formation enthalpy of CuInSe_2_ is lower than that of CuGaSe_2_^42–44^. Therefore, as GGI decreases, the CIGSe formation enthalpy becomes relatively smaller, allowing for the formation of larger grains as there is sufficient energy available for recrystallization^45^. Additionally, towards the GGI value at which c/a = 2, the tetragonal distortion becomes reduced, reducing also the lattice strain and thus, leading to larger grain sizes. As a result, the grain size exhibits a peak value around c/a = 2. These findings highlight an intriguing fact that within a single sample, the grain size is influenced by variations in GGI, rather than differences observed between samples with varying GGIs. Understanding the relationship between grain size and GGI in CIGSe films is crucial for advancing the development of more efficient and stable solar cells. Further research, involving measurements of the lattice parameter directly instead of relying solely on calculations based on GGI, is necessary to obtain a comprehensive understanding of the underlying mechanisms.

### 3D Simulation

**Figure 10:**
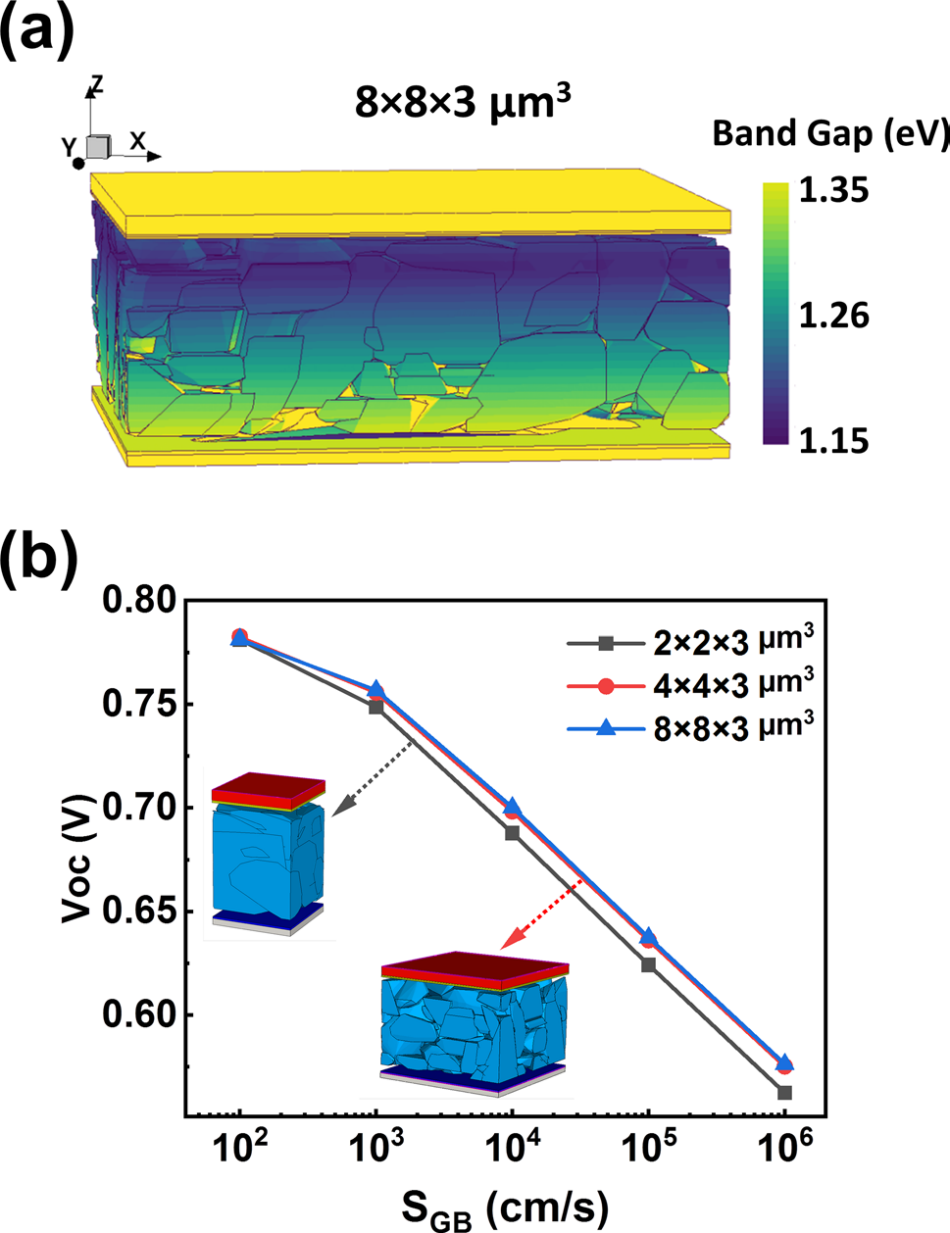
(**a**) 3D simulation model of 8 × 8 × 3 µm^3^ using the GGI depth profiling data to calculate the applied band gap; the color bar on the right represents the band gap values. (**b**) V_OC_ plot as a function of the grain boundary recombination S_GB_ for different model sizes: 2 × 2 × 3 µm^3^ model (black squares), 4 × 4 × 3 µm^3^ model (red circles), and 8 × 8 × 3 µm^3^ model (blue triangles). The V_OC_ results for the 8 × 8 × 3 µm^3^ and 4 × 4 × 3 µm^3^ models display similar trends, suggesting that they are comparable in representing the sample behavior. In contrast, the 2 ×  2 ×  3 µm^3^ model diverges in values, indicating that this smaller model size may not be sufficient for accurate simulation outcomes.

As a proof of concept, we used the three-dimensional structure model of the CIGSe sample for first optoelectronic device simulations. This was combined with a one-dimensional bandgap profile by employing the GGI depth profile in Fig. 9a and using the formula E_g CIGSe_ = E_g CIS_ × (1 − x) + E_g CGS_ × x − bx(1 − x). Here x stands for the GGI, E_g CIS_ is the bandgap of CuInSe_2_, E_g CGS_ is the bandgap of CuGaSe_2_, and b is the bowing parameter, which is 0.2 for CIGSe^46^. The color-coded bandgap profile can be seen in Fig. 10a.

Three models with dimensions of the same device with sizes 2 × 2 × 3 µm^3^, 4 × 4 × 3 µm^3^, and 8 × 8 × 3 µm^3^ were used for the simulations where all models shared the same central point. Photovoltaic simulations were conducted using Sentaurus TCAD, calculating the open-circuit voltage (V_OC_) under 1 sun 1.5G illumination. In each model, the grain boundary recombination velocity (S_GB_) was varied. All other physical parameters are fixed as detailed in supplementary Table S1.

As seen in Fig. 10b, the V_OC_ decreases with increasing S_GB_. This is expected as a higher number of carriers recombine at grain boundaries, leading to a decrease in V_OC_. At small S_GB_ values, the results from each model are similar. However, for S_GB_ values at or above 1000 cms^−1^, the V_OC_ of the 2 × 2 × 3 µm^3^ model shows a more pronounced decrease in V_OC_ compared to those from the 4 × 4 × 3 µm^3^ and 8 × 8 × 3 µm^3^ models. The effect of grain boundary recombination is based on the grain distribution. Obviously, averaging is sufficiently similar for the 4 × 4 × 3 µm^3^ and 8 × 8 × 3 µm^3^ models. However, for the 2 × 2 × 3 µm^3^ model, averaging around the central point is not sufficiently representative. Averaging then will become position dependent. The effect of grain boundary recombination is influenced by grain distribution. In 1D models or under simpler conditions, the recombination effect scales with grain boundary density. However, in complex 2D model or 3D model, not just the grain boundary density but also the position and shape of the grain boundaries became significant factors. Particular, areas with similar electron and hole densities have stronger recombination at the grain boundaries^47^. Interestingly, the selected 2 × 2 × 3 µm^3^ model, although having a lower grain boundary density compared to the other sizes, exhibits a more pronounced effect from the grain boundaries. This is due to the grain boundaries in the 2 × 2 × 3 µm^3^ model being located in areas with high recombination activity, causing the effects to be locally significant rather than averaged out like in the other models. Therefore, for more accurate simulations, it is essential to choose a model size that effectively represents the distribution of the grain boundaries.

Such results can vary depending on the chosen location, indicating that the 2 × 2 × 3 µm^3^ range might not be suitable for a comprehensive simulation of the entire structure, which aligns with the findings from the previous structural analysis section. Considering the simulation runtimes of 8984 s, 28,576 s, and 100,799 s for each model respectively, it's inferred that the 4 × 4 × 3 µm^3^ model is the most appropriate one for 3D simulation of this CIGSe sample.

## Conclusion

In this study, we introduced a novel approach to reconstructing the 3D structure of high-efficiency CIGSe samples using EBSD data. By employing the 2-opt algorithm and the convex-hull algorithm, we successfully reduced the data volume while maintaining the accuracy of the grain structure reconstruction. The resulting 3D model demonstrated improved surface area accuracy compared to traditional voxel-based models. Our findings highlight the potential of this approach in enhancing our understanding of complex material structures.

By the analysis of the reconstructed 3D structure, we gained valuable insights into the CIGSe samples. By effectively representing the grain distribution, a model size of 4 × 4 × 4 µm^3^ was identified as a suitable compromise between sample representation and data economy. Utilizing GOS, we examined the strain within individual grains in a three-dimensional context, providing further understanding of the material properties. A significant inverse relationship between grain size and GGI, suggesting a close correlation with the crystallographic c/a ratio, was revealed. Grain surface to volume ratio could largely be described by a constant factor which may be an indication of grain recrystallization during CIGSe growth. Additionally, leveraging the analyzed structural characteristics, we successfully crafted an electronic 3D simulation. Through this, we discerned the importance of appropriate range selection in creating an efficient simulation that aptly represents the entirety of the sample.

The present research highlights the significance of 3D analysis in advancing our understanding of CIGSe and polycrystalline materials. The proposed methodology shows great potential for conducting comprehensive and detailed analyses of various polycrystalline materials, enabling a deeper understanding of their 3D structure and its impact on material properties. Moreover, the insights gained from this study can contribute to the broader fields of photovoltaics and materials science, thereby facilitating the development of more efficient and sustainable technologies.

### Supplementary Information

Supplementary Information.

## Data Availability

The data and code used in this study are available upon request from the corresponding author.

## References

[1] Louat NP (1974). On the theory of normal grain growth. ACTA Metall..

[2] Groeber M, Ghosh S, Uchic MD, Dimiduk DM (2008). A framework for automated analysis and simulation of 3D polycrystalline microstructures. Part 1: Statistical characterization. Acta Mater..

[3] Li W, Yan X, Aberle AG, Venkataraj S (2019). Effect of sodium diffusion on the properties of CIGS solar absorbers prepared using elemental Se in a two-step process. Sci Rep.

[4] Krause M, Nikolaeva A, Maiberg M (2020). Microscopic origins of performance losses in highly efficient Cu(In, Ga)Se2 thin-film solar cells. Nat. Commun..

[5] Colombara D, Stanbery BJ, Sozzi G (2023). Revani diffusion model in Cu(In, Ga)S_e_e. J. Mater. Chem. A.

[6] Ren X, Yang Z, Yang D (2016). Modulating crystal grain size and optoelectronic properties of perovskite films for solar cells by reaction temperature. Nanoscale.

[7] Si Z, Yuan Q, Wang S (2022). Effects of Na doping on the distribution of elements and the formation of back surface field in CIGS absorption layer. Appl. Phys. A Mater. Sci. Process..

[8] Sun Q, Asqardoust S, Sarmah A, Jain MK (2022). Elastoplastic analysis of AA7075-O aluminum sheet by hybrid micro-scale representative volume element modeling with really-distributed particles and in-situ SEM experimental testing. J. Mater. Sci. Technol..

[9] DeMott R, Haghdadi N, Kong C (2021). 3D electron backscatter diffraction characterization of fine α titanium microstructures: Collection, reconstruction, and analysis methods. Ultramicroscopy.

[10] Pirgazi H (2019). On the alignment of 3D EBSD data collected by serial sectioning technique. Mater. Charact..

[11] Gholinia A, Curd ME, Bousser E (2020). Coupled broad ion beam-scanning electron microscopy (BIB–SEM) for polishing and three dimensional (3D) serial section tomography (SST). Ultramicroscopy.

[12] Gholinia A, Brough I, Humphreys J, Bate P (2012). A 3D FIB investigation of dynamic recrystallization in a Cu-Sn bronze. Materials Science Forum.

[13] Burnett TL, Kelley R, Winiarski B (2016). Large volume serial section tomography by Xe Plasma FIB dual beam microscopy. Ultramicroscopy.

[14] Polonsky AT, Lenthe WC, Echlin MP (2020). Solidification-driven orientation gradients in additively manufactured stainless steel. Acta Mater..

[15] Louca K, Abdolvand H (2021). Accurate determination of grain properties using three-dimensional synchrotron X-ray diffraction: A comparison with EBSD. Mater. Charact..

[16] Viganò N, Ludwig W, Batenburg KJ (2014). Reconstruction of local orientation in grains using a discrete representation of orientation space. J. Appl. Crystallogr..

[17] McDonald SA, Burnett TL, Donoghue J (2021). Tracking polycrystal evolution non-destructively in 3D by laboratory X-ray diffraction contrast tomography. Mater. Charact..

[18] Viganò N, Tanguy A, Hallais S (2016). Three-dimensional full-field X-ray orientation microscopy. Sci. Rep..

[19] DeMott R, Haghdadi N, Liao X (2021). 3D characterization of microstructural evolution and variant selection in additively manufactured Ti-6Al-4 V. J Mater. Sci..

[20] Francis T, Rottmann PF, Polonsky AT (2021). Multimodal 3D characterization of voids in shock-loaded tantalum: Implications for ductile spallation mechanisms. Acta Mater..

[21] Winiarski B, Gholinia A, Mingard K (2021). Correction of artefacts associated with large area EBSD. Ultramicroscopy.

[22] Stechmann G, Zaefferer S, Konijnenberg P (2016). 3-Dimensional microstructural characterization of CdTe absorber layers from CdTe/CdS thin film solar cells. Sol. Energy Mater. Sol. Cells.

[23] Abou-Ras D, Caballero R, Fischer CH (2011). Comprehensive comparison of various techniques for the analysis of elemental distributions in thin films. Microsc. Microanal..

[24] Kodalle T, Greiner D, Brackmann V (2019). Glow discharge optical emission spectrometry for quantitative depth profiling of CIGS thin-films. J. Anal. At. Spectrom..

[25] Möller B, Glaß M, Misiak D, Posch S (2016). MiToBo - A toolbox for image processing and analysis. J. Open Res. Softw..

[26] Preibisch S, Saalfeld S, Tomancak P (2009). Globally optimal stitching of tiled 3D microscopic image acquisitions. Bioinformatics.

[27] Johnson DS, McGeoch LA, Aarts EHL, Lenstra JK (1997). The traveling salesman problem: A case study in local optimization. Local Search in Combinatorial Optimization.

[28] Jia X, Liu Z, Han Y (2022). Sphericity and roundness for three-dimensional high explosive particles by computational geometry. Comput. Part Mech..

[29] Šedivý O, Dake JM, Krill CE (2017). Description of the 3D morphology of grain boundaries in aluminum alloys using tessellation models generated by ellipsoids. Image Anal. Stereol..

[30] Šedivý O, Brereton T, Westhoff D (2016). 3D reconstruction of grains in polycrystalline materials using a tessellation model with curved grain boundaries. Philos. Mag..

[31] Fátima Vaz M, Fortes MA (1988). Grain size distribution: The lognormal and the gamma distribution functions. Scr. Metall..

[32] Liu W, Lian J, Aravas N, Münstermann S (2020). A strategy for synthetic microstructure generation and crystal plasticity parameter calibration of fine-grain-structured dual-phase steel. Int. J. Plast..

[33] Wang C, Liu G, Wang G, Xue W (2007). Three-dimensional grain size distribution: Comparison of an analytical form under a topology-related rate equation with computer simulations and experimental data. Mater. Sci. Eng. A.

[34] Ashby MF (1993). Materials Selection in Mechanical Design.

[35] Kessler J, Chityuttakan C, Lu J (2003). Cu(In, Ga)S_e_2 thin films grown with a Cu-poor/rich/poor sequence: Growth model and structural considerations. Prog. Photovolt. Res. Appl..

[36] Jariwala S, Sun H, Adhyaksa GWP (2019). Local crystal misorientation influences non-radiative recombination in halide perovskites. Joule.

[37] Li K, Yang P (2017). The formation of strong 100 texture by dynamic strain-induced boundary migration in hot compressed Ti-5Al-5Mo-5V-1Cr-1Fe alloy. Metals (Basel).

[38] Gabor AM, Tuttle JR, Albin DS (1994). High-efficiency CuIn_x_Ga_1-x_Se_2_ solar cells made from (In_x_, G_a1__−_x_)2_Se_3_ precursor films. Appl. Phys. Lett..

[39] Schleussner SM, Törndahl T, Linnarsson M (2012). Development of gallium gradients in three-stage Cu(In, Ga)Se_2_ co-evaporation processes. Prog. Photovolt. Res. Appl..

[40] Abou-Ras D, Caballero R, Kaufmann CA (2008). Impact of the Ga concentration on the microstructure of Culn_1-__−__x_GaxSe_2_. Phys. Status Solidi Rapid Res. Lett..

[41] Raghuwanshi M, Cadel E, Duguay S (2017). Influence of Na on grain boundary and properties of Cu(In, Ga)Se_2_ solar cells. Prog. Photovolt. Res. Appl..

[42] Ider M (2020). Determination of the gibbs formation energy of CuGaSe_2_ by EMF method. Int. J. Electrochem. Sci..

[43] Kim S, Kim WK, Kaczynski RM (2005). Reaction kinetics of CuInSe_2_ thin films grown from bilayer InSe/CuSe precursors. J. Vac. Sci. Technol. A Vacuum Surfaces Film.

[44] Kim WK, Payzant EA, Kim S (2008). Reaction kinetics of CuGaSe_2_ formation from a GaSe/CuSe bilayer precursor film. J. Cryst. Growth.

[45] Rodriguez-Alvarez H, Weber A, Lauche J (2013). Formation of CuInSe_2_ and CuGaSe_2_ thin-films deposited by three-stage thermal co-evaporation: A real-time X-ray diffraction and fluorescence study. Adv. Energy Mater..

[46] Scheer R, Schock HW (2011). Chalcogenide Photovoltaics: Physics, Technologies, and Thin Film Devices.

[47] Gloeckler M, Sites JR, Metzger WK (2005). Grain-boundary recombination in Cu(In, Ga)Se2 solar cells. J. Appl. Phys..

