# Supplementary material for: A new approach to three-dimensional microstructure reconstruction of a polycrystalline solar cell using high-efficiency Cu(In,Ga)Se2
Source: Sci Rep. 2024 Jan 23;14:2036. doi: 10.1038/s41598-024-52436-2 (PMC10805891; doi:10.1038/s41598-024-52436-2)
Supplement: Supplementary file 1 — Supplementary Information. [file 41598_2024_52436_MOESM1_ESM.docx]

Electronic Supplementary Material for:

**A new approach to three-dimensional microstructure reconstruction of a polycrystalline solar cell using high efficiency Cu(In,Ga)Se_2_**

Chang-Yun Song^1*^, Matthias Maiberg^1^, Heiko Kempa^1^, Wolfram Witte^2^, Dimitrios Hariskos^2^, Daniel Abou-Ras^3^, Birgit Moeller^4^, Roland Scheer^1^ and Ali Gholinia^5^

1. Martin Luther University Halle-Wittenberg, Institute of Physics, Von-Danckelmann-Platz 3, 06120 Halle (Saale), Germany
2. Zentrum für Sonnenenergie- und Wasserstoff-Forschung Baden-Württemberg (ZSW), Meitnerstr. 1, 70563 Stuttgart, Germany
3. Helmholtz-Zentrum Berlin für Materialien und Energie GmbH, Hahn-Meitner-Platz 1, 14109 Berlin, Germany
4. Martin Luther University Halle-Wittenberg, Institute of Computer Science, von-Seckendorff-Platz 1, 06120 Halle (Saale), Germany
5. Department of Materials, The University of Manchester, Manchester M13 9PL, United Kingdom


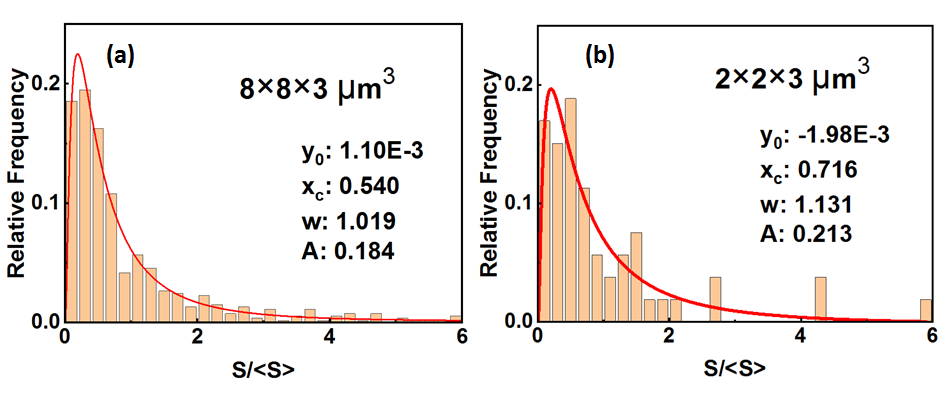


Figure S 1 *Normalized surface area distribution of grains S/<S> for the 8* × *8* × *3* *μm^3^ sample (a) and for the 2* × *2* × *3* *μm^3^ sample (b), with LogNormal fits (red line) and fitting parameter values. 2* × *2* × *3* *μm^3^* *sample, the fitting parameter values show a significant difference compared to other samples.*


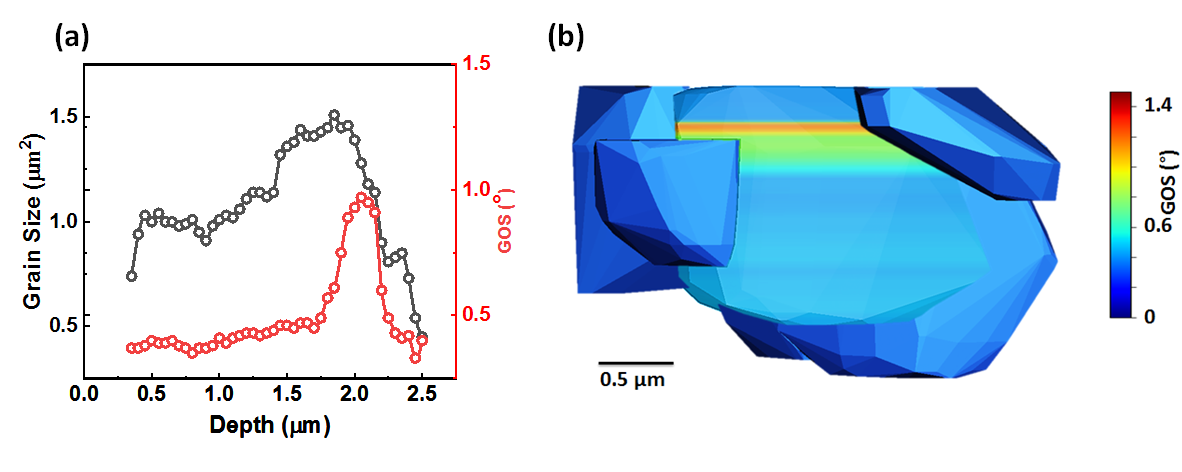


Figure S 2 *(a) Grain size (black) and GOS (red) of middle grain. (b) 3D representation of two grains with large GOS (color coded) in certain regions and surrounding grains. The color code represents the GOS.*

| **Layer**  [thickness (*μ*m)] | **Parameter** | | | | | | | |
| --- | --- | --- | --- | --- | --- | --- | --- | --- |
|  | **Doping density** (cm^-3^) | **Mobility**  (cm^2^/Vs) | | **Lifetime**  (ns) | | **Density of state** (cm^-3^) | | **Radiative recombination** (cm^3^/s) |
|  |  | electron | hole | electron | hole | electron | hole |  |
| **ZnO:Al**  **[0.25]** | 8×10^19^ | 20 | 20 | 10 | 0.01 | 4×10^18^ | 9×10^18^ | - |
| **(Zn,Mg)O**  **[0.025]** | 6×10^16^ | 3 | 0.9 | 3.2×10^6^ | 77 | 3×10^18^ | 1.7×10^18^ | - |
| **CdS**  **[0.03]** | 2×10^16^ | 20 | 20 | 1 | 0.001 | 4×10^18^ | 9×10^18^ | - |
| **CIGSe**  **[2.8]** | 1.7×10^15^ | 31 | 28 | 43 | 780 | 7×10^17^ | 1.5×10^19^ | 7.7×10^-10^ |

Table S 1 Fixed simulation parameter values according to each layer.
